# Power difference in a χ^2^ test vs generalized linear mixed model in the presence of missing data – a simulation study

**DOI:** 10.1186/s12874-020-00936-w

**Published:** 2020-03-02

**Authors:** Mary L. Miller, Denise J. Roe, Chengcheng Hu, Melanie L. Bell

**Affiliations:** Department of Epidemiology and Biostatistics, University of Arizona, PO Box 245163, Tucson, AZ 85724 USA

**Keywords:** Complete-case, Missing data, Binary data, Generalized linear mixed model, Chi-squared test, Power, Relative bias, Longitudinal

## Abstract

**Background:**

Longitudinal randomized controlled trials (RCTs) often aim to test and measure the effect of treatment between arms at a single time point. A two-sample χ^2^ test is a common statistical approach when outcome data are binary. However, only complete outcomes are used in the analysis. Missing responses are common in longitudinal RCTs and by only analyzing complete data, power may be reduced and estimates could be biased. Generalized linear mixed models (GLMM) with a random intercept can be used to test and estimate the treatment effect, which may increase power and reduce bias.

**Methods:**

We simulated longitudinal binary RCT data to compare the performance of a complete case χ2 test to a GLMM in terms of power, type I error, relative bias, and coverage under different missing data mechanisms (missing completely at random and missing at random). We considered how the baseline probability of the event, within subject correlation, and dropout rates under various missing mechanisms impacted each performance measure.

**Results:**

When outcome data were missing completely at random, both χ2 and GLMM produced unbiased estimates; however, the GLMM returned an absolute power gain up to from 12.0% as compared to the χ^2^ test. When outcome data were missing at random, the GLMM yielded an absolute power gain up to 42.7% and estimates were unbiased or less biased compared to the χ2 test.

**Conclusions:**

Investigators wishing to test for a treatment effect between treatment arms in longitudinal RCTs with binary outcome data in the presence of missing data should use a GLMM to gain power and produce minimally unbiased estimates instead of a complete case χ2 test.

## Background

### Complete binary outcomes

Association of response and treatment at a single time point in a randomized clinical trial (RCT) with binary outcomes can be analyzed by using a χ^2^test of association, methods of moments generalized estimating equations (GEE), or likelihood based generalized linear mixed models (GLMM). When the data are complete, estimates of the treatment effect are unbiased [1] for any of these methods. However, in longitudinal RCTs, complete data rarely exist. A recent review found that 95% of RCTs in the top 4 medical journals had reported some amount of missing outcome data [2]. Potential consequences of missing data include decreased precision, lower power, and biased estimates [3], which can lead to improper inferences of between and within arm effects.

### Mechanisms for missing data

Rubin [4] has defined the probability mechanisms for missing data into three categories: missing completely at random (MCAR), missing at random (MAR), and not missing at random (MNAR). Under MCAR, the probability of missingness does not depend on the unobserved or observed data. An example of MCAR data would be if a researcher lost part of their data set to a computer crash. For MAR, the probability of data being missing depends on the set of prior observed responses but is unrelated to the responses that would have been obtained. An example of MAR data would be that a participant misses an appointment because he or she was too sick (or healthy) at the previous appointment and didn’t want to attend the current one. MNAR data are data where the probability of missingness is related to the missing data itself, even after taking account of observed data.

### Statistical approaches to longitudinal binary data with missing data

Weighted GEE (wGEE) or multiple imputation (MI) with GEE are valid options to analyze incomplete longitudinal binary data, as long as models are correctly specified. The unbiasedness of a χ^2^test and GEE is based on the assumption that missing outcome data are MCAR [1], but this strong assumption is often unrealistic in longitudinal RCTs. A more appropriate assumption for missingness is MAR, where unbiased estimates can be obtained by GLMM, wGEE, and MI with GEE [1].

Researchers have studied the effects of MCAR and MAR missing data on the analyses of binary data from longitudinal RCTs for GEE, extensions to GEE including MI with GEE, and GLMM. A simulation study done by Beunckens et al. showed that in small to moderate sample size (*n* = 50 per treatment arm) with MAR data, MI with GEE was less biased and more precise compared to wGEE [5]. Lipkovich et al. demonstrated that under MAR, MI with GEE resulted in less biased estimates, higher power, and smaller Type I error rates compared to unweighted GEE and GLMM [6]. A more recent paper by Liu and Zhan simulated repeated binary responses with missing data under MCAR and MAR, and compared the Type I error rate and power obtained from GLMM (full and pseudo restricted likelihood), unweighted GEE, and several MI approaches. Under MAR, pseudo-likelihood GLMM performed better than GEE and MI with logistic regression in terms of controlling Type I error rate and power [7]. Additionally, bias under GLMM and MI with logistic regression was well controlled.

Although the effects of missing data on power and between arm estimates in longitudinal RCTs with binary outcomes have been investigated with statistical analyses such as MI with GEE, GEE, wGEE, and GLMM, little research has been done to compare power and bias of treatment effects between the χ2 method and GLMM. We chose χ2for our analysis because it is the most common statistical analysis for between arm treatment effect in RCTs with binary outcomes [2]. The χ2 is the most commonly used statistical analysis of longitudinal binary RCT outcome data because we desire the unadjusted between arm treatment effect at time point j. However, the χ2 test ignores the within subject correlation that is inherent to longitudinal RCT data and excludes any outcome data that are missing from the analysis. In contrast, generalized linear mixed models (GLMM), which are an extension of generalized linear models, account for the within-subject association by introduction a subset of regression coefficients (random effects) that vary randomly across individuals. Inferences of fixed effects are conditional on random effects and have subject specific interpretations. We used GLMM (which produces subject-specific treatment effects by default) as a comparative method instead of GEE (which produces marginal treatment effects) because it is known that under MAR, non-likelihood based GEE will yield biased estimates of the mean response [1].

We hypothesized that power would be higher and estimates of the between arm treatment effect would be less biased for pseudo-likelihood GLMM compared to the χ^2^ test when missing data are present. Our rationale comes from a simulation study done by Ashbeck et al., where the effects of missing data at a fixed time point in a longitudinal RCT with continuous outcomes was studied. Power and bias were compared between a complete case two sample t-test and mixed model for repeated measures (MMRM) in the presence of missing data. When data were MCAR, estimates remained unbiased for both tests; however, MMRM had an absolute power gain up to 12%. MMRM outperformed t-test when data were MAR in terms of less biased estimates and higher power [8]. We suspect that power and bias obtained from complete case χ^2^ and GLMM will behave in a similar manner as presented by Ashbeck et al.

This paper follows best practices for simulation studies as outlined by Morris et al. [9] and is organized as follows. Section 2 describes the aims, data generation, statistical analysis, and performance measures used in this paper. Analytic results of performance measures from each simulation scenario under each missing mechanism are presented in Section 3. Section 4 discusses results and concluding remarks are presented in Section 5.

## Methods

### Aims

The primary aims of this simulation study were to evaluate the impact of MCAR and MAR data on the a) power to detect a treatment effect between two treatment arms at the final time point and b) estimated treatment effect between arms obtained from χ2 and GLMM analyses. We investigated the sensitivity of our results to the control arm proportion and correlation between repeated measures.

### Data generation

A two-arm randomized controlled trial with a 1:1 treatment allocation ratio was used to generate three complete repeated binary outcomes for each individual, where the third measurement was the primary time point for analysis. Let Y_ij_ be an observed binary outcome for the i^th^ (i = 1, …,N) subject at the j = 1,..,3 occasion in a longitudinal RCT with treatment variable X_i_ = 0 (control) or 1 (treatment), where the number of visits was fixed and the same for all participants. Responses between subjects were assumed to be independent, but responses within subjects were correlated. Let p_ij_ be the probability of the event for the i^th^ person at the j^th^ time point and Y_ij_ = 1 represent that a positive outcome has occurred; conversely, Y_ij_ = 0 indicates the participant experienced a negative outcome.

At baseline, a random p_i1_ (baseline probability of event-also known as prevalence rate) for each individual was generated from a Beta(a, b) distribution. While p_i1_ varied between participants, average p_i1_ across all participants was 0.1 or 0.5, regardless of treatment arm (i.e. p_i1_ = 0.1 or 0.5). Next, a random y_i1_ was generated from a Binomial(1, p_i1_) distribution. The expected value of Y_i1_ is ${p}_{i1}=\frac{a}{a+b}$ and correlation between two repeated measures is ρ $=\frac{1}{1+a+b}$. The values for *a* and *b* were fixed to produce a prevalence rate of 0.1 or 0.5 and ρ of 0.3 or 0.7. The first N/2 participants were assigned to the treatment and the latter half were assigned to the control arm.

For each subsequent time point j (j = 2, 3), the probability of Y_ij_ = 1 was derived from the following formula:

1 $$ {p}_{ij}={p}_{i\left(j-1\right)}+0.05\ast {X}_i $$

**Fig. 1:**
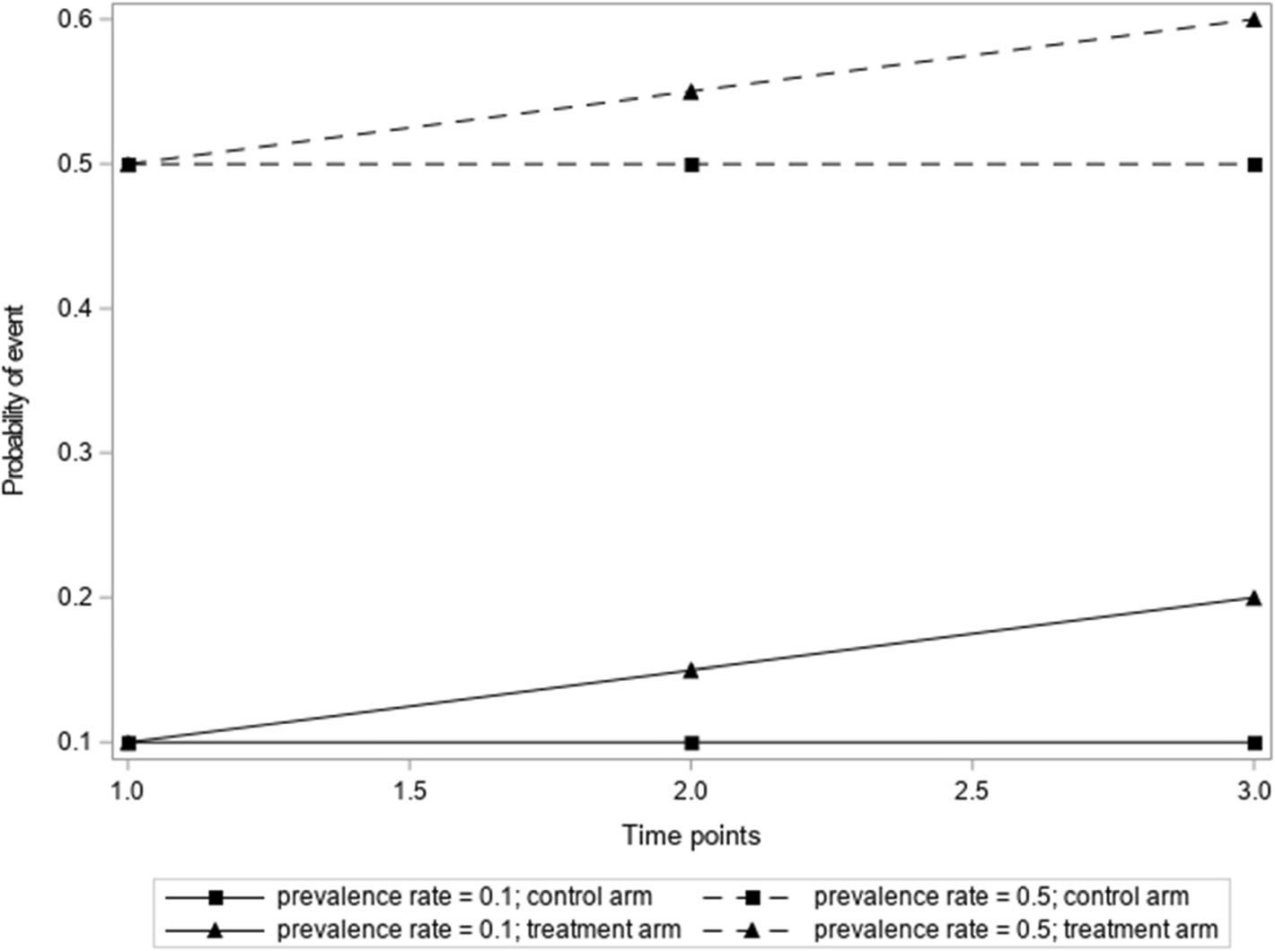
Simulated trajectories of probability of Y_ij_ for p_i1_ = 0.1 (solid line) and p_i1_ = 0.5 (dashed line)

The fixed coefficient 0.05 induced a linear increase of p_i1_ between arms at j. A random y_ij_ was then generated from a binomial (1, p_ij_) distribution. The simulation produced a fixed end-of-study (j = 3) risk difference of 0.10. Probabilities generated from [1] for the treatment arm increased linearly but remained constant for the control arm (see Fig. 1). In order for the χ^2^ test to have 80% power to detect an end-of-study risk difference of 0.1, a total sample size of 398 (p_i1_ = 0.1) or 776 (p_i1_ = 0.5) was needed, assuming no continuity correction. Additionally, we generated data under the null hypothesis of no risk difference at any time point. We used the same data generation technique as described above to generate Y_ij_, except p_ij_ was the same for each time point. Therefore, there was no difference in the within and between arm treatment effect at any time point.

### Missing data generation

In order to investigate the effects of missing data on power and bias of estimates obtained from χ^2^ and GLMM analyses, observations from complete data sets at end-of-study were deleted under MCAR and MAR mechanisms. Rates of missingness were broken into two categories: equal and unequal. Equal dropout rates had 30% of observations deleted per arm. Unequal dropout rates were further separated into two subcategories – *unequal dropouts 1* (20% missing from control arm and 40% missing from treatment arm) and *unequal dropouts 2* (40% missing from control arm and 20% missing from treatment arm). Data sets under each p_i1_ and ρ combination were constructed under the following scenarios:

1. *No missing data.* One thousand complete data sets were generated under each p_i1_ (0.1 and 0.5) with corresponding ρ (0.3 and 0.7) using the data generation method described in the *Data Generation* section.
2. *Missing completely at random (MCAR).* The probability of missingness was unrelated to previous or current values of Y and treatment arm. Observations from each complete dataset at the third time point were selected for deletion via simple random sampling.
3. *Missing at random (MAR).* Outcomes at time point three were deleted based on the previous value of Y (Y_i2_) and treatment arm. The MAR mechanism was modeled as
  $$ logit\left(\Pr \left({y}_{i3}=.|{y}_{i2},{X}_i\right)\right)={\gamma}_1+{\gamma}_2{y}_{i2}+{\gamma}_3{X}_i $$

where Pr(*y*_*i*3_ = .| *y*_*i*2_, *X*_*i*_) is the probability of an observation being deleted at the third time point. Therefore, the probability of missingness depended on the previous observation (*y*_*i*2_ = 0 *or* 1) and treatment assignment (*X*_*i*_ = 0 *or* 1). The coefficient *γ*_2_ was fixed prior to data generation while *γ*_1_ and *γ*_3_ were varied to achieve desired rates of missingness. After obtaining Pr(*y*_*i*3_ = .| *y*_*i*2_, *X*_*i*_), a Bernoulli random variable was generated to determine if the participant had dropped out at the third time point. We varied the values of *γ*_2_ to determine its impact on power and bias of treatment effect.

- *MAR1* - The value of *γ*_2_ was set to 1.5. Individuals who responded positively at time point 2 (y_i2_ = 1) would have 4.5 times the odds of having a missing observation at time point 3 compared to those who responded poorly (y_i2_ = 0). Therefore, a positive response at time point 2 indicates that the participant’s third observation was more likely to be deleted.
- *MAR2* - The value of *γ*_2_ was set to − 1.5. Individuals who responded with y_i2_ = 1 have 0.22 times the odds of having a missing response at time point 3 compared to individuals who responded y_i2_ = 0. Thus, participants who responded negatively at the prior time point were more likely to have their third observation deleted from the data set.

MNAR data were excluded from the data generating process. When data are assumed to be MNAR, focus shifts from primary analysis (χ2 and GLMM) to a sensitivity analysis, which can require complex models such as selection or pattern mixture models [10]. This is not the focus of this paper and examples of the analysis for longitudinal binary data with MNAR missing outcomes can be found elsewhere [11].

### Statistical analyses

We tested the null hypothesis of no treatment effect between treatment arms at the final time point and estimated the treatment effect through a log-odds ratio (log-OR), which captured the likelihood of Y_i3_ = 1 in the treatment arm compared to the control. The log-ORs associated with χ2 were calculated from a 2 × 2 contingency table and through GLMM with the use of a contrast. Estimates obtained from the complete data sets were defined as the “true” treatment effect and used for comparison purposes.

### χ2 test for association

Let Y_ij_ = 1 represent the occurrence of event for the i^th^ patient at time point j and Y_ij_ = 0 otherwise. For each replicate, observed counts of responses for each treatment arm at time point j = 3 were organized into a 2 × 2 contingency table, which was used to calculate a Pearson χ2 test statistic and other measures (explained below in the *Performance Measures* section).

### Generalized linear mixed model

Let Y_ij_ be a binary response for the i^th^ individual at time point j. Then the response probability Pr(Y_ij_ = 1|b_i_) can be modeled as follows:

$$ logit\left(\Pr \left({Y}_{ij}=1|{b}_i\right)\right)={\beta}_1+{\beta}_2{X}_i+{\beta}_3{t}_{ij}+{\beta}_4{t}_{ij}{X}_i+{b}_i $$

where *t*_*ij*_ represent the continuous time measurement for the i^th^ individual (i = 1, …,N) at the j^th^ time point (j = 1,..,3), *b*_*i*_ is the between-subject random effect, and *β*_4_ is the log-OR between arms at time point j. It is assumed that ${b}_i\sim N\Big(0,{\sigma}_b^2$) and denotes the variability between individuals in the baseline probability.

### Performance measures

We ran 1000 simulations for all combinations of p_i1_ and ρ, under each missing mechanism and dropout rate. From each analysis, we estimated the treatment effect (log-OR), the Wald statistic and its associated *p*-value, standard error, and 95% confidence intervals. We then calculated the mean treatment effect as the average log-OR (sum of log-OR/1000). We used this information to construct the following performance measures for each statistical test.

- **Power and Type I error.** Given that the data were simulated under the alternative (risk difference = 0.10) and null (risk difference = 0) hypotheses, we assessed power and type I error rate, which was calculated as the percentage of *p*-values < 0.05 (# of p-values < 0.05/1000 * 100%) under each hypothesis.
- **Treatment effect and relative bias.** Treatment effects from χ2 test were calculated from 2 × 2 contingency tables while estimates from the GLMM were obtained by the use of a contrast. Relative bias was defined as $\frac{\overline{\hat{\theta}}-{\theta}_T}{\theta_T}\ast 100\%$, where *θ*_*T*_ is the mean log-odds ratio derived from the complete data set and $\overline{\hat{\theta}}$ is the mean log-odds ratio obtained from the incomplete data sets.
- **Coverage** was defined as the percentage of confidence intervals that contained *θ*_*T*_, the mean log-OR obtained from the complete data set.
- **Model-based and empirical standard error**, which measure the precision of the treatment effect. Model SE was calculated as the square root of the average of the variances from all 1000 replicates and empirical SE was derived by the traditional standard deviation calculation with no distinction between replicates. We expect these standard errors to be equivalent.

The main performance measures were relative bias and power for the log-odds comparing treatment versus control. We consider a statistical method superior if it has higher power (compared to other method) and minimal bias. We will tolerate small biases if power gained is substantial. While there is no definite range of bias that is acceptable, we will consider an estimate minimally biased if relative bias falls between ±10% [7]. If a situation arises where power gained is moderate but estimates are clearly biased, preference of test will default to the one that reduces bias.

All simulations and analyses were performed in SAS version 9.4 and are available through the online Appendix. The χ2 method and its associated performance measures were implemented via PROC FREQ. Model parameters (covariance matrix and fixed effects) were estimated using restricted pseudo-likelihood (RPL) GLMM with random intercept and compound symmetric variance-covariance matrix through PROC GLIMMIX with the ESTIMATE statement.

We chose a compound symmetric model as it is appropriate when the mean response derived from a generalized linear mixed model depends on the population parameters β and a single random effect b_i_. We believe that the use of an unstructured model would not substantially change the results and would unnecessarily increase computation time due to the increase in matrix parameters.

Other likelihood estimation techniques, such as Laplace and adaptive quadrature, are available in SAS. However, we chose RPL because it is the default estimation method used in PROC GLIMMIX [12]. Although other estimation techniques may be used that maximize the full-likelihood, these techniques can be computationally intensive because the integrals are approximated numerically. Since we are not maximizing a true likelihood function, a Wald statistic was used to test for treatment effect instead of the likelihood ratio test.

## Results

For data simulated under the alternative hypothesis, summary of performance measures for χ2 and GLMM methods for each prevalence and correlation combination under each missing mechanism and rates of missingness are presented in Tables 1, 2, 3, 4. We present the Type I error rates in Table 5.

**Table 1:** Summary of empirical estimates of log-OR and relative bias (%), power (%), coverage (%), model standard error and empirical standard error derived from *χ*^2^ method and GLMM test with compound symmetric variance-covariance matrix from 1000 simulations with a total sample size *N* = 398

	*p*_*i*1_ = 0.1, *ρ* = 0.3				
	Log-odds (% bias)	Power (%)^c^	Coverage (%)^c^	Model SE^c^	Empirical SE^c^
**Complete**					
*χ*^2a^	0.831 (0)	82.0	95.0	0.300	0.300
GLMM^b^	0.886 (0)	85.7	96.8	0.301	0.283
**MCAR**					
**Equal drop out (30% drop out in each arm)**					
*χ*^2^	0.840 (1.1)	65.6	95.0	0.362	0.371
GLMM	0.894 (1.0)	73.2	96.9	0.355	0.329
**Unequal dropouts 1 (20% drop out in control, 40% drop out in treatment)**					
*χ*^2^	0.814 (−2.0)	65.8	95.4	0.356	0.356
GLMM	0.886 (0.1)	72.8	96.4	0.353	0.326
**Unequal dropouts 2 (40% drop out in control, 20% drop out in treatment)**					
*χ*^2^	0.841 (1.3)	62.5	95.5	0.375	0.380
GLMM	0.886 (0)	71.5	97.1	0.362	0.326
**MAR1 (observations more likely to be deleted if prior observation was positive)**					
Equal drop out (30% drop out in each arm)					
*χ*^2^	0.921 (10.9)	69.2	95.2	0.386	0.395
GLMM	0.949 (7.2)	76.5	96.7	0.363	0.337
**Unequal dropouts 1 (20% drop out in control, 40% drop out in treatment)**					
*χ*^2^	0.834 (0.5)	62.8	94.9	0.376	0.386
GLMM	0.928 (4.8)	74.4	96.8	0.359	0.331
**Unequal dropouts 2 (40% drop out in control, 20% drop out in treatment)**					
*χ*^2^	0.991 (19.3)	74.4	95.7	0.402	0.407
GLMM	0.959 (8.3)	76.7	97.4	0.369	0.340
**MAR2 (observations less likely to be deleted if prior observation was positive)**					
**Equal drop out (30% drop out in each arm)**					
*χ*^2^	0.791 (−4.8)	65.5	95.0	0.353	0.358
GLMM	0.858 (−3.1)	71.1	97.1	0.354	0.327
**Unequal dropouts 1 (20% drop out in control, 40% drop out in treatment)**					
*χ*^2^	0.848 (2.1)	70.9	94.7	0.351	0.362
GLMM	0.871 (−1.6)	71.9	96.7	0.353	0.328
**Unequal dropouts 2 (40% drop out in control, 20% drop out in treatment)**					
*χ*^2^	0.749 (−9.9)	56.2	93.0	0.361	0.369
GLMM	0.853 (−3.7)	67.8	95.7	0.359	0.337

^a^Independent two-sample χ2 test for test of treatment effect at time point 3

^b^Generalized linear mixed model with compound symmetric variance-covariance matrix, contrast used to estimate treatment effect at time point 3

^c^Range of Monte Carlo SE: Power (0.011–0.016); Coverage (0.005–0.008); Model SE (0.0004–0.002); Empirical SE (0.006–0.009)

**Table 2:** Summary of empirical estimates of log-OR and relative bias (%), power (%) and MC standard error, coverage (%), model standard error and empirical standard error derived from *χ*^2^ method and GLMM test with compound symmetric variance-covariance matrix from 1000 simulations with a total sample size *N* = 398

	*p*_*i*1_ = 0.1, *ρ* = 0.7				
	Log-odds (% bias)	Power (%)^c^	Coverage (%)^c^	Model SE^c^	Empirical SE^c^
**Complete**					
*χ*^2 a^	0.792 (0)	78.2	95.8	0.301	0.295
GLMM^b^	0.944 (0)	84.7	98.1	0.335	0.292
**MCAR**					
**Equal drop out (30% drop out in each arm)**					
*χ*^2^	0.798 (0.7)	62.3	95.0	0.362	0.367
GLMM	0.953 (0.9)	73.5	98.4	0.384	0.328
**Unequal dropouts 1 (20% drop out in control, 40% drop out in treatment)**					
*χ*^2^	0.796 (0.5)	64.6	95.3	0.357	0.354
GLMM	0.961 (1.8)	74.9	98.3	0.381	0.326
**Unequal dropouts 2 (40% drop out in control, 20% drop out in treatment)**					
*χ*^2^	0.803 (1.4)	60.2	95.4	0.373	0.372
GLMM	0.946 (0.2)	72.2	98.0	0.390	0.329
**MAR1 (observations more likely to be deleted if prior observation was positive)**					
**Equal drop out (30% drop out in each arm)**					
*χ*^2^	1.032 (30.2)	73.8	93.3	0.424	0.427
GLMM	1.075 (13.9)	83.3	97.5	0.400	0.341
**Unequal dropouts 1 (20% drop out in control, 40% drop out in treatment)**					
*χ*^2^	0.842 (6.3)	58.1	95.3	0.402	0.406
GLMM	1.016 (7.6)	79.1	98.0	0.392	0.336
**Unequal dropouts 2 (40% drop out in control, 20% drop out in treatment)**					
*χ*^2^	1.194 (50.3)	83.1	89.7	0.455	0.463
GLMM	1.117 (18.3)	85.0	97.2	0.408	0.346
**MAR2 (observations less likely to be deleted if prior observation was positive)**					
**Equal drop out (30% drop out in each arm)**					
*χ*^2^	0.690 (−12.9)	55.6	93.7	0.339	0.336
GLMM	0.879 (−7.0)	65.3	98.2	0.380	0.325
**Unequal dropouts 1 (20% drop out in control, 40% drop out in treatment)**					
*χ*^2^	0.826 (4.3)	69.7	95.8	0.340	0.337
GLMM	0.935 (−1.0)	72.3	98.4	0.380	0.326
**Unequal dropouts 2 (40% drop out in control, 20% drop out in treatment)**					
*χ*^2^	0.577 (−27.2)	37.3	89.1	0.342	0.346
GLMM	0.831 (−12.0)	58.4	97.5	0.382	0.328

^a^Independent two-sample χ2 test for test of treatment effect at time point 3

^b^Generalized linear mixed model with compound symmetric variance-covariance matrix, contrast used to estimate treatment effect at time point 3

^c^Range of Monte Carlo SE: Power (0.011–0.016); Coverage (0.004–0.010); Model SE (0.0002–0.003); Empirical SE (0.007–0.010)

**Table 3:** Summary of empirical estimates of log-OR and relative bias (%), power (%) and MC standard error, coverage (%), model standard error and empirical standard error derived from *χ*^2^ method and GLMM test with compound symmetric variance-covariance matrix from 1000 simulations with a total sample size *N* = 776

	*p*_*i*1_ = 0.5, *ρ* = 0.3				
	Log-odds (% bias)	Power (%)^c^	Coverage (%)^c^	Model SE^c^	Empirical SE^c^
**Complete**					
*χ*^2 a^	0.396 (0)	78.1	93.6	0.145	0.144
GLMM^b^	0.507 (0)	75.6	97.5	0.199	0.171
**MCAR**					
**Equal drop out (30% drop out in each arm)**					
*χ*^2^	0.392 (−1.0)	61.6	95.0	0.174	0.175
GLMM	0.508 (0.3)	61.7	97.3	0.223	0.198
**Unequal dropouts 1 (20% drop out in control, 40% drop out in treatment)**					
*χ*^2^	0.404 (2.0)	61.5	95.3	0.176	0.176
GLMM	0.516 (1.8)	63.9	97.1	0.224	0.197
**Unequal dropouts 2 (40% drop out in control, 20% drop out in treatment)**					
*χ*^2^	0.394 (−0.3)	63.0	96.3	0.175	0.170
GLMM	0.510 (0.6)	65.4	98.0	0.224	0.193
**MAR1 (observations more likely to be deleted if prior observation was positive)**					
**Equal drop out (30% drop out in each arm)**					
*χ*^2^	0.405 (2.3)	63.6	95.9	0.173	0.172
GLMM	0.510 (0.7)	64.7	97.7	0.223	0.193
**Unequal dropouts 1 (20% drop out in control, 40% drop out in treatment)**					
*χ*^2^	0.314 (−20.6)	43.9	93.2	0.145	0.174
GLMM	0.529 (4.3)	66.6	97.8	0.223	0.194
**Unequal dropouts 2 (40% drop out in control, 20% drop out in treatment)**					
*χ*^2^	0.495 (25.2)	80.2	91.0	0.175	0.174
GLMM	0.495 (−2.4)	60.6	97.4	0.224	0.194
**MAR2 (observations less likely to be deleted if prior observation was positive)**					
**Equal drop out (30% drop out in each arm)**					
*χ*^2^	0.406 (2.7)	63.9	94.5	0.176	0.177
GLMM	0.514 (1.4)	65.8	97.8	0.224	0.198
**Unequal dropouts 1 (20% drop out in control, 40% drop out in treatment)**					
*χ*^2^	0.496 (25.4)	79.7	91.4	0.179	0.176
GLMM	0.494 (−2.6)	60.0	97.7	0.225	0.197
**Unequal dropouts 2 (40% drop out in control, 20% drop out in treatment)**					
*χ*^2^	0.311 (−21.3)	41.4	93.2	0.177	0.177
GLMM	0.530 (4.5)	66.9	97.6	0.224	0.196

^a^Independent two-sample χ2 test for test of treatment effect at time point 3

^b^Generalized linear mixed model with compound symmetric variance-covariance matrix, contrast used to estimate treatment effect at time point 3

^c^Range of Monte Carlo SE: Power (0.013–0.016); Coverage (0.004–0.009); Model SE (< 0.0001–0.001); Empirical SE (0.003–0.004)

**Table 4:** Summary of empirical estimates of log-OR and relative bias (%), power (%) and MC standard error, coverage (%), model standard error and empirical standard error derived from *χ*^2^ method and GLMM test with compound symmetric variance-covariance matrix from 1000 simulations with a total sample size *N* = 776

	*p*_*i*1_ = 0.5, *ρ* = 0.7				
	Log-odds (% bias)	Power (%)^c^	Coverage (%)^c^	Model SE^c^	Empirical SE^c^
**Complete**					
*χ*^2 a^	0.394 (0)	77.2	95.5	0.145	0.145
GLMM^b^	0.624 (0)	78.7	97.2	0.234	0.208
**MCAR**					
**Equal drop out (30% drop out in each arm)**					
*χ*^2^	0.397 (0.7)	63.3	95.5	0.174	0.175
GLMM	0.631 (1.1)	70.0	97.6	0.259	0.225
**Unequal dropouts 1 (20% drop out in control, 40% drop out in treatment)**					
*χ*^2^	0.396 (0.5)	61.5	95.1	0.176	0.176
GLMM	0.633 (1.4)	70.7	97.0	0.261	0.229
**Unequal dropouts 2 (40% drop out in control, 20% drop out in treatment)**					
*χ*^2^	0.392 (−0.6)	61.3	96.0	0.175	0.170
GLMM	0.628 (0.7)	71.5	97.7	0.260	0.220
**MAR1 (observations more likely to be deleted if prior observation was positive)**					
**Equal drop out (30% drop out in each arm)**					
*χ*^2^	0.453 (14.8)	74.3	93.2	0.173	0.173
GLMM	0.672 (7.8)	77.5	96.9	0.258	0.226
**Unequal dropouts 1 (20% drop out in control, 40% drop out in treatment)**					
*χ*^2^	0.257 (−34.9)	30.1	87.4	0.174	0.173
GLMM	0.635 (1.7)	72.8	97.4	0.259	0.225
**Unequal dropouts 2 (40% drop out in control, 20% drop out in treatment)**					
*χ*^2^	0.649 (64.6)	95.3	71.0	0.177	0.178
GLMM	0.709 (13.7)	82.1	95.6	0.259	0.227
**MAR2 (observations less likely to be deleted if prior observation was positive)**					
**Equal drop out (30% drop out in each arm)**					
*χ*^2^	0.374 (−5.2)	54.7	94.9	0.179	0.177
GLMM	0.586 (−6.1)	63.6	97.5	0.260	0.224
**Unequal dropouts 1 (20% drop out in control, 40% drop out in treatment)**					
*χ*^2^	0.583 (47.9)	89.9	82.5	0.183	0.183
GLMM	0.632 (1.2)	70.6	97.4	0.262	0.227
**Unequal dropouts 2 (40% drop out in control, 20% drop out in treatment)**					
*χ*^2^	0.164 (−58.3)	14.7	75.7	0.180	0.177
GLMM	0.541 (−13.3)	54.3	96.6	0.261	0.221

^a^Independent two-sample χ2 test for test of treatment effect at time point 3

^b^Generalized linear mixed model with compound symmetric variance-covariance matrix, contrast used to estimate treatment effect at time point 3

^c^Range of Monte Carlo SE: Power (0.007–0.016); Coverage (0.005–0.014); Model SE (< 0.0001–0.001); Empirical SE (0.003–0.005)

**Table 5:** Summary of empirical Type I error rates (%) derived from *χ*^2^ method and GLMM test with compound symmetric variance-covariance matrix from 1000 simulations with a total sample size *N* = 398 (p_i1_ = 0.1) and *N* = 776 (p_i1_ = 0.5)

	p_i1_ = 0.1, ρ = 0.3^c^	p_i1_ = 0.1, ρ = 0.7^c^	p_i1_ = 0.5, ρ = 0.3^c^	p_i1_ = 0.5, ρ = 0.7^c^
**Complete**				
*χ*^2 a^	6.2	6.6	5.1	4.5
GLMM^b^	4.0	3.2	3.4	2.8
**MCAR**				
**Equal drop out (30% drop out in each arm)**				
*χ*^2 a^	6.2	5.8	4.7	4.8
GLMM^b^	3.3	3.0	2.9	2.7
**Unequal dropouts 1 (20% drop out in control, 40% drop out in treatment)**				
*χ*^2 a^	6.0	5.3	4.6	5.0
GLMM^b^	3.7	2.4	3.5	1.7
**Unequal dropouts 2 (40% drop out in control, 20% drop out in treatment)**				
*χ*^2 a^	4.5	6.0	4.7	3.7
GLMM^b^	3.8	2.5	3.1	2.1
**MAR1 (observations more likely to be deleted if prior observation was positive)**				
**Equal drop out (30% drop out in each arm)**				
*χ*^2 a^	4.8	4.7	5.1	4.6
GLMM^b^	3.0	2.2	2.7	2.5
**Unequal dropouts 1 (20% drop out in control, 40% drop out in treatment)**				
*χ*^2 a^	4.7	6.7	6.7	19.4
GLMM^b^	2.7	1.8	2.6	2.3
**Unequal dropouts 2 (40% drop out in control, 20% drop out in treatment)**				
*χ*^2 a^	5.3	8.3	9.5	27.6
GLMM^b^	2.9	2.5	2.7	3.0
**MAR2 (observations less likely to be deleted if prior observation was positive)**				
**Equal drop out (30% drop out in each arm)**				
*χ*^2 a^	4.6	5.5	5.0	5.3
GLMM^b^	3.6	2.8	3.1	2.9
**Unequal dropouts 1 (20% drop out in control, 40% drop out in treatment)**				
*χ*^2 a^	6.3	10.1	9.6	27.3
GLMM^b^	3.8	3.3	2.3	3.1
**Unequal dropouts 2 (40% drop out in control, 20% drop out in treatment)**				
*χ*^2 a^	5.9	8.3	7.5	19.5
GLMM^b^	3.6	4.1	2.9	2.5

^a^Independent two-sample χ2 test for test of treatment effect at time point 3

^b^Generalized linear mixed model with compound symmetric variance-covariance matrix, contrast used to estimate treatment effect at time point 3

^c^Range of Monte Carlo SE for Type I error rate: (0.005–0.008); (0.004–0.010); (0.005–0.010); (0.004–0.014)

### MCAR

The GLMM method, on average, performed better than the χ2 test in terms of power gain and reduction of bias of the estimates under MCAR. In most missing scenarios, coverage was at or above the expected 95%, and empirical and model-based standard errors were roughly equivalent for both methods. Type I error rates ranged from 1.7 to 6.2%, where all rates obtained from the GLMM analyses fell under the anticipated value of 5% (1.7–3.8%). While estimates derived from both 2 × 2 tables and GLMM under MCAR were unbiased, a maximum absolute power gain of 12.0% was obtained from GLMM method over the χ2 test.

### MAR

When outcome data were MAR, the GLMM outperformed χ2 in terms of absolute power gain, controlling the Type I error rate, and reduction of bias. We saw absolute power gain peak at 42.7% (p_i1_ = 0.5, ρ = 0.7, *MAR1*, *unequal dropout 1*). While not quite as high, the absolute power gained by using the GLMM test over χ2 when correlation was lower (ρ = 0.3) was still substantial at 25.5% (p_i1_ = 0.5, *MAR1*, *unequal dropout 1*). The GLMM analysis had the lowest Type I error rate (1.8%) and none of the values were above 5%. The χ2 test inflated the Type I error rate under unequal dropout rates, with the most severe cases (maximum 27.6%) occurring when p_i1_ = 0.5. Biased estimates were obtained from the GLMM, ranging from − 13.3 to 18.3%. However, the χ2 test was unable to control bias as well as the GLMM, with relative bias ranging from − 58.5 to 64.6%.

### MAR, low prevalence

Under MAR simulation where the prevalence rate was low (p_i1_ = 0.1), the advantage of the GLMM test over the χ2 was apparent when ρ = 0.7, with an absolute power gain of 21.1%, while only a 1.0% power gain occurred when ρ = 0.3. In addition to higher power, the GLMM estimates were typically unbiased while log-ORs calculated from the 2 × 2 tables were not. The magnitude of the relative bias increased as ρ did, while the directionality of the bias (over or underestimated treatment effect) was affected by the missing mechanisms (*MAR1* vs *MAR2*) and dropout rates, with relative bias ranging from − 12.0 to 18.0% from the GLMM and − 27.2 to 50.3% from the χ2 method. Treatment effects from both methods were overestimated when ρ = 0.7 under *MAR1, equal* and *unequal dropout1* and underestimated under *MAR2, equal* and *unequal dropout2*. Biased estimates affected the percentage of confidence intervals that contained the true log-ORs, specifically when the analysis was performed with the χ2 method, with coverage as low as 89.1%. Standard errors were approximately equal under both methods. The GLMM better controlled the Type I error rates (1.8 to 3.0%) compared to the χ2 test (4.7 to 8.3%). See Tables 1, 2 and 5.

### MAR, high prevalence

As baseline probability of event increased from 0.1 to 0.5, so did the need to use GLMM to control for bias and increase power. Relative bias was near acceptable limits, ranging from − 13.3 to 13.7%, while relative bias from χ2 analysis ranged from − 58.5 to 64.3%. The bounds of these intervals occurred when ρ = 0.7 and dropout rates were unequal. The power gained from the GLMM analysis over χ2 was substantial, with an absolute power difference of 42.7% when ρ = 0.7 (*MAR1, unequal dropouts 1*). In general, power increased by using the GLMM over χ2. However, there were scenarios where power was lost, specifically under *MAR1 unequal dropouts 2* and *MAR2 unequal dropouts 1*. We caution the reader to not focus solely on power gain and instead look at the whole picture, as treatment effects obtained from the χ2 analysis are severely biased, coverage is not adequate, and type I error are severely inflated for these scenarios. See Tables 3, 4, and 5.

## Discussion

We compared bias and power of the GLMM and χ2 methods with binary outcomes from longitudinal RCT data in the presence of missing data. We found that the GLMM, on average, produced the least biased estimates, had higher power to detect a treatment effect compared to the χ2 test without increasing Type I error rate, and coverage was at or above the anticipated 95%. We saw similar conclusions presented by Liu et al. [7] when discussing relative bias and coverage between a GLMM analysis compared to GEE for longitudinal binary outcomes with MCAR and MAR data.

Standard errors within and between each method were roughly equivalent when the prevalence rate was low, but the GLMM standard errors were higher than the χ2 standard errors when the data were simulated under a high prevalence rate. We would expect the standard errors from the GLMM analysis to be smaller than the standard errors calculated from the χ2 method, as the GLMM accounts for the within-subject correlation. However, estimates derived from the GLMM analysis have a subject-specific interpretation, which are larger in absolute value than the marginal estimates [1] obtained from the χ2 test. The standard error is a function of the estimate; therefore it is not unexpected that the standard errors associated with the GLMM were larger than those obtained from the χ2 analysis. That said, it is not appropriate to compare standard errors between the two tests due to the interpretation of these estimates.

It should be noted that the GLMM model is a more flexible model that can include covariates; therefore, we might expect it to perform better than the χ2 method, which does not include covariates. However, the primary analysis of the between arm treatment effect in a longitudinal RCT is typically unadjusted. We felt it was appropriate to consider the χ2 model as the standard to compare the GLMM method to, as it is the most commonly used statistical analysis in RCTs [2].

### MCAR

As expected, unbiased estimates of treatment effect were produced from both GLMM and χ2 tests for all parameter combinations of p_i1_ and ρ when missing outcome data were MCAR. Given that the observations selected for deletion were chosen at random, both complete case and mixed model methods provided accurate log-ORs [1]. GLMM was able to incorporate correlation between measurements (via random effects), resulting in higher power compared to the χ2 test.

### MAR

Estimates of log-ORs at the final time point calculated from a 2 × 2 table were biased under both MAR1 and MAR2. Treatment effects obtained from the GLMM analysis were less biased and few exceeded the relative bias threshold of ±10%.Extreme bias occurred when correlation between measurements was high. We note that the directionality of bias was not consistent in MAR1 and MAR2 when prevalence rate fluctuated between 0.1 and 0.5.However, this does not change our recommendation that GLMM be used as the primary statistical method to estimate treatment effect. While in practice we cannot adequately state why an observation is missing unless detailed follow up occurred, we can clearly see that GLMM minimizes bias under MAR for all dropout scenarios.

Substantial power was gained by using GLMM over χ2 in most missing scenarios, with values ranging from 1.0 to 42.7%. However, GLMM lost power compared to χ2 when p_i1_ = 0.5 and a) *MAR1* and *dropouts unequal 1* and b) *MAR2* and *dropouts unequal 2*. This increase in power from the χ2 test directly corresponds to the inflated treatment effect. We cannot recommend a test that overestimates the effect of a treatment, no matter how powerful the test may be.

### Strengths and limitations

Liu and Zhan [7] reported on the bias and power gained comparing a variety of statistical methods, such as logistic regression, GEE, and GLMM, for the analysis of repeated binary data in the presence of missing data. However, they did not consider the impact of different directions of dropout under MAR, which we consider an important strength to our simulation study.

A limitation to this study is the interpretation of treatment effects from each method. Odds ratios associated with χ2 analysis have population-specific interpretations while those obtained from GLMM are subject-specific. It is known that subject-specific estimates will be larger in absolute value than population-specific estimates [1]. Relative bias of population-specific estimates from GLMM may not be equivalent to what was obtained from subject-specific analysis. As is true for all simulation studies, we could not investigate every possible scenario. We investigated one possible trajectory for the treatment and control arms. We did not consider how other estimation methods (e.g., Laplace and adaptive quadrature) and tests for treatment effects (e.g., likelihood ratio test) impact bias and other performance measures. Finally, we did not investigate covariance patterns beyond compound symmetry.

A possible extension to this area of study would be to include the outcome of event at baseline (Y_i1_) as a covariate in the analysis of repeated binary data with missing outcomes, sometimes referred to as ANCOVA. Jiang et al [13]. evaluated the effect of baseline adjusted and unadjusted analyses on longitudinal binary data in the presence of missing data. Analyses included logistic regression with last observation carried forward, GLMM, GEE, wGEE, and MI with GEE. On average, adjusted analyses yielded less biased estimates and increased power compared to their unadjusted counterparts. Future analyses of incomplete repeated binary data may include a logistic regression model and a GLMM, both with adjustment for baseline outcome.

A limitation, raised by a reviewer, is that we did not consider Bayesian models for the analysis of longitudinal binary RCT outcome data. We acknowledge that while Bayesian analyses may result in higher power and less bias of estimates compared to frequentist methods, they are less commonly used in analysis of longitudinal RCT. We leave the investigation of this and other potential factors to future research.

## Conclusion

In this paper, we considered the impact of missing outcome data on power, bias, coverage, and standard errors obtained from the χ2 test and generalized linear mixed model. Based on the results from these simulations, we recommend the use of a GLMM for the primary analysis of longitudinal binary data in the presence of missing data, which was robust against missing data and provided less bias and higher power than χ2 methods.

## Supplementary information

**Additional file 1.** Supplemental SAS simulation and analysis.

## Data Availability

This was a simulation study, therefore there are no real data. However, simulation code can be found in the appendix (see Additional file 1).

## Abbreviations

GEE: Generalized estimating equations
GLMM: Generalized linear mixed models
log-OR: Log-odds ratios
MAR: Missing at random
MCAR: Missing completely at random
MI: Multiple imputation
MMRM: Mixed model for repeated measures
MNAR: Not missing at random
RCT: Randomized clinical trial
RPL: Restricted pseudo-likelihood
wGEE: weighted GEE
