# Supplementary material for: Power difference in a χ2 test vs generalized linear mixed model in the presence of missing data – a simulation study
Source: BMC Med Res Methodol. 2020 Mar 2;20:50. doi: 10.1186/s12874-020-00936-w (PMC7053142; doi:10.1186/s12874-020-00936-w)
Supplement: Supplementary file 1 — Additional file 1. Supplemental SAS simulation and analysis. [file 12874_2020_936_MOESM1_ESM.docx]

| Table 1: Code for generation of correlated longitudinal binary data |
| --- |
| /*MACRO FOR CORRELATED BINARY DATA  mu = prevalence rate (pi1 = 0.1 or 0.5)  rho = correlation (0.3 or 0.7)  risk_diff = difference in pij at time point j. Set to 0.05  numrep = number of replicates (set to 1000)  npergroup = number of individuals per treatment arm (depends on pi1 and risk_diff at j = 3)  seed = seed value for random number generation  */  **%macro** betabin(mu =, rho = , risk_diff = , numrep = , npergroup = , seed = );  %let a = %sysevalf(&mu.*(1-&rho.)/&rho.);  %let b = %sysevalf((1-&rho.)*(1-&mu.)/&rho);  %let time = 3;  %let risk_diff = &risk_diff;  keep rep id p j t trt y;  %let people = &npergroup*2;  call streaminit(&seed);  do rep = **1** to &numrep;  do id = **1** to &people;  p = rand("Beta", &a, &b);  if id <= &npergroup then trt = **1**; else trt = **0**;    do j = **1** to &time;  if j ne **1** then p = min(p + &risk_diff*trt, **1**);  y = rand("Binomial", p, **1**);  t = j;  output;  end;  end;  end;  run;  **%mend** betabin; |

Online Appendix

| Table 2: Generation of MAR data using data generated from Table 1 |
| --- |
| /*  MISSING AT RANDOM DATA GENERATION  file = name of complete data set  mar = name of outfile  c, e = tuning parameters (gamma1, gamma3 in paper)  d = fixed a priori (gamma2 in paper; fixed at 1.5 or -1.5)  */  **%macro** mar(file = , mar = , c = , d = , e =);  proc sort data = &file; by rep id descending trt j; run;  data &mar;  set &file;  y2 = lag(y);  if j ne **3** then y2 = **.**;  if j = **3** then p_miss = exp(&c + &d*y2 + &e*trt)/(**1**+exp(&c + &d*y2 + &e*trt));    call streaminit(**123**);  if p_miss ne **.** then y3_missing = rand("Bernoulli", p_miss);  if y3_missing = **1** then y = **.**;  run;  **%mend**; |

| Table 3: Analysis of longitudinal correlated binary data at final time point |
| --- |
| /*  CHI SQUARE ANALYSIS  FILE = NAME OF CORRELATED BETA-BINOMIAL DATA (COMPLETE OR MISSING DATA)  POWER = DATA SET THAT WILL CONTAIN P-VALUES  ESTIMATES = DATA SET THAT WILL CONTAIN LOG-ODD RATIOS  FREQ = DATA SET THAT CONTAINS 2X2 CONTINGENCY TABLES  */  **%macro** chisq(file = , power = , estimates = , FREQ = );  proc sort data = &FILE; by rep descending trt descending y; run; *SORT DATA BY REP, THEN TRT = 1 TO 0 AND Y = 1 TO 0;  ods graphics off;  ods exclude all;  proc freq data = &FILE order = data;  by rep;  where j = **3**;  table trt*y/chisq relrisk chisq nocol norow nopercent;  ods output ChiSq = &POWER; *FOR POWER ANALYSIS;  ods output RelativeRisks = &ESTIMATES; *TO DERIVE LOG-OR, COVERAGE;  ods output crosstabfreqs = &FREQ; *TO CALCULATE AVERAGE MODEL SE;  run;  ods exclude none;  **%mend**;  /*  GENERALIZED LINEAR MIXED MODEL (SUBJECT SPECIFIC) ANALYSIS  file = longitudinal correlated binary data (complete or missing) in LONG form  power = name of data set that contains results from ESTIMATE statement  */  **%macro** glmmOR(file = , j = , power = );  *LINK = LOGIT --> OR;  proc sort data=&file; by rep id j; run;  ods graphics off;  ods exclude all;  proc glimmix data = &file;  by rep;  class id;  model y(descending) = trt j trt*j/dist= binary link=logit solution;  random intercept/subject = id type=cs;  estimate 'OR trt vs ctrl: time pt 3' trt **1** trt*j **3**/exp cl;  nloptions technique = none;  ods output Estimates = &power;  run;  ods exclude none;  **%mend**; |
